# Remote Mentorship Using Video Conferencing as an Effective Tool to Strengthen Laboratory Quality Management in Clinical Laboratories: Lessons From Cambodia

**DOI:** 10.9745/GHSP-D-20-00128

**Published:** 2020-12-23

**Authors:** Grant Donovan, Siew Kim Ong, Sophanna Song, Nayah Ndefru, Chhayheng Leang, Sophat Sek, Patricia Sadate-Ngatchou, Lucy A. Perrone

**Affiliations:** a Department of Global Health, Schools of Public Health and Medicine, University of Washington, Seattle, WA, USA.; b International Training and Education Center for Health, Cambodia, Phnom Penh, Cambodia.; c International Training and Education Center for Health, Department of Global Health, Schools of Public Health and Medicine, University of Washington, Seattle, WA, USA.

## Abstract

**Background:**

Providing professional development opportunities to staff working in clinical laboratories undergoing quality improvement programs can be challenged by limited funding, particularly in resource-limited countries such as Cambodia. Using innovative approaches such as video conferencing can connect mentors with practitioners regardless of location. This study describes and evaluates the methods, outputs, and outcomes of a quality improvement program implemented in 12 public hospital laboratories in Cambodia between January 2018 and April 2019. The program used mixed intervention methods including both in-person and remote-access training and mentorship.

**Methods:**

Training outputs were quantified from the activity reports of program trainers and mentors. Program outcomes were measured by pre- and postimplementation audits of laboratory quality management system conformity to international standards. Variations in improved outcomes were assessed in relation to the time spent by laboratory personnel in video conference training and mentoring activity. An additional cross-sectional comparison described the difference in final audit scores between participating and nonparticipating laboratories.

**Results:**

Laboratories significantly improved their audit scores over the project period, showing significant improvement in all sections of the ISO 15189 standard. Pre- and postaudit score differences and laboratory personnel participation time in remote mentoring activities showed a strong monotonic relationship. Average input per laboratory was 6,027±2,454 minutes of participation in video conference activities with mentors. Audit scores of participating laboratories were significantly higher than those of laboratories with no quality improvement program.

**Conclusion:**

Laboratories improved significantly in ISO 15189 conformity following structured laboratory quality management systems training supported by remote and on-site mentoring. The correlation of laboratory participation in video conference activities highlights the utility of remote video conferencing technology to strengthen laboratories in resource-limited settings and to build communities of practice to address quality improvement issues in health care. These findings are particularly relevant in light of the COVID-19 pandemic.

## Abstract (precis)

This program to strengthen laboratory quality management systems in Cambodia demonstrated significant improvements in conformity to ISO 15189 standards in participating laboratories, correlating with laboratory participation time in video conference training activities led by quality improvement mentors over the program implementation period.

## Teaser

**Key Findings:**

- Utilization of a mixed-methods intervention design combining remote and in-person training, accompanied by close mentorship, contributed to successful implementation of quality management systems in participating laboratories.
- Laboratory participation time in video conference training activities correlated with better quality management system management and improved conformity to the ISO 15189 standard for medical laboratories.

**Key Implications:**

- When identifying budget and policy priorities for health, policy makers should consider the beneficial impact of a sustained human resources training and mentorship program on laboratory quality improvement and service delivery efforts.
- Policy makers should particularly consider the potential efficiency and effectiveness of remote-access telementoring and teleconferencing to support online communities of practice for laboratory professionals because improved connectivity and knowledge sharing between professionals are essential for quality service delivery in a laboratory system.

## INTRODUCTION

Development of strong laboratory quality management systems (LQMS) is a key component of strengthening health systems for improved health outcomes and disease surveillance in resource-limited countries, and it requires standardization and strategic planning.^1^ ^–^ ^3^ ISO 15189 accreditation, which is the international standard for medical laboratory quality, provides standardization of LQMS requirements with a strong technical foundation for health, safety, and conformity.^4^ ^,^ ^5^ These standards are stringent, however, and have required a variety of approaches for laboratories with different resource availability and levels of development to achieve them.^5^ ^,^ ^6^ In Cambodia, a national effort to meet International Health Regulations and improve health services has culminated in an expansive national laboratory system to meet the diagnostic and surveillance needs of the country at both the national and provincial levels.^7^ The country has adopted national standards for medical laboratories, integrated a system of external quality assessments through private and public partnerships, and developed a national laboratory information system to improve surveillance and care, but structured quality improvement (QI) programs are limited to only a subset of laboratories. Expansion of these quality management training programs to meet international standards of quality was recommended in a series of laboratory assessments carried out between 2013 and 2016.^7^ ^,^ ^8^ One of these assessments measured 11 indicators of laboratory capacity, identifying a low average score of 36% in 22 laboratories, with indicators of LQMS averaging only 47% due to a lack of quality management systems, trained quality assurance managers, or continuous improvement practices.^8^

The implementation of structured, stepwise programs to improve quality management systems in national and provincial laboratories has been integral to improving laboratory quality and capacity in Cambodia.^8^ ^,^ ^9^ In 2001, the U.S. Centers for Disease Control and Prevention partnered with the Cambodia Ministry of Health (MOH) to implement the Strengthening Laboratory Management Toward Accreditation program using the Stepwise Laboratory Improvement Process Towards Accreditation (SLIPTA) audit tool, supported by quality management training and mentorship by trained QI professionals (BMLS Cambodia, unpublished presentation, 2018).^9^ International Training and Education Center for Health (I-TECH’s) QI program began in 2014 and was intended to expand access to LQMS training and implementation coaching nationally, delivered through a complementary package of training, mentorship, and technical assistance to MOH for national laboratory policy and guideline development. Success of these programs in Cambodia and globally has shown the impact that structured and mentored LQMS programs can have in resource-limited health care systems, holding promise for other such programs in Cambodia in the future.^8^ ^,^ ^9^

However, delivery of professional training and close mentorship for laboratories undergoing QI programs remains challenged by geographical and economic constraints. These challenges have prompted the use of modern video conferencing technologies to expand access to consultation from quality management professionals to distal facilities. Studies have shown that the use of these technologies, collectively known as telementoring, is an efficient and cost-effective tool to provide seasoned or specialized expertise to health professionals in remote or resource-limited medical facilities and has an impact on professional behavior and knowledge, as well as health outcomes.^10^ ^–^ ^12^ One recent program in Southeast Europe used monthly mentorship through telecommunication to improve laboratory quality in 5 countries and demonstrated measurable progress within the 6 laboratories supported.^13^ One of the most successful models of telementoring, Project ECHO (Extension for Community Healthcare Outcomes), has recently been expanded to laboratory strengthening, and the institute is now partnering with at least 5 major professional laboratory institutions to provide laboratory training and mentorship communities of practice globally.^14^ Research demonstrating the effectiveness of this model of remote training and mentoring for laboratory strengthening is limited, however, prompting a need for quantitative research.

Delivery of professional training and close mentorship for laboratories undergoing QI programs is challenging due to geographical and economic constraints.

### Program Description

During the initial phase (Phase 1) of this project in Cambodia in 2014, a group of 12 participating laboratories received training and mentored technical support to implement an LQMS according to the newly published World Health Organization (WHO) Laboratory Quality Stepwise Implementation (LQSI) tool.^15^ Evaluation of that phase of the program indicated that consistent on-site mentoring in the local language with a stepwise action plan enhanced staff knowledge of LQMS implementation towards meeting the ISO 15189 standard, without interrupting regular laboratory services.^8^ The successful results of this training and mentoring approach led to an initiative by MOH to expand laboratory access to LQMS training and mentorship that prioritized implementation of national standards of quality nationwide.

These priorities triggered the need for additional innovative approaches in 2017 for the second phase (Phase 2) of the QI program in Cambodia. At the time, Cambodia did not have a national standard of quality, a standard tool for laboratory assessment, or a law to enforce laboratory quality in the public or private sectors. To address this issue, the Cambodia Laboratory Quality Management System Checklist for Accreditation (CamLQMS) was developed and adopted by the MOH Bureau of Medical Laboratory Services (BMLS) as a tool for national auditors to assess laboratory quality during on-site performance audits. The CamLQMS tool was modeled on the WHO-AFRO SLIPTA tool, which is aligned with the ISO 15189 standard.^7^ ^,^ ^8^ ^,^ ^16^ Between July 2017 and April 2019, the Phase 2 QI program used the CamLQMS tool to track laboratory progress toward meeting ISO 15189 accreditation standards, while using a combination of training, on-site and remote mentorship, and advocacy.

Phase 2 of the QI program directed a set of technical approaches and interventions at both the national and regional levels to strengthen the interconnectivity and collaboration between laboratories in the tiered laboratory system for improved public health and clinical functions. At the national level, the program worked to address gaps in the legal and regulatory framework and documentation concerning the establishment of national quality and safety standards. At the facility level, primary activities encompassed the design and delivery of job-specific, competency-based education and training to quality assurance officers (QAOs) and laboratory managers selected from the Phase 1 cohort of the 12 national and regional clinical laboratories. Facility-based staff were trained in operational quality management and provided regular mentoring support through on-site technical assistance and telementoring consultations. In January of 2018, I-TECH Cambodia partnered with MOH-BMLS to conduct a baseline CamLQMS assessment of participating laboratories, followed by a national dissemination meeting to discuss findings and develop recommendations. These recommendations included a series of 11 training workshops to improve the LQMS operational practices of QAOs and laboratory managers and to eliminate deficiencies identified during the audits.^17^ LQMS trainings were designed using adult learning principles and accepted pedagogy to improve learner comprehension and competency through a combination of theoretical and practical learning methods oriented toward health professionals.^18^ These trainings consisted of large-group formal instruction interspersed with several focused and interactive sessions over 2–5 days, as well as smaller laboratory-based training workshops held regionally to emphasize practical proficiency in technical skills. Although subject matter content focused primarily on quality assurance and operational management, laboratory managers and QAOs were also provided with organizational leadership skill-building activities.

Phase 2 of the QI program also included close mentorship of laboratory staff by trained laboratory quality mentors. As described previously,^8^ mentors were technically experienced laboratory professionals, trained in QI, proficient in both English and Khmer, and employed in the project full time. Mentors periodically visited laboratories to deliver individualized training and coaching to each of the 12 laboratories and to address the gaps identified in the baseline audits; however, for Phase 2 of the project, the majority of mentorship and supportive coaching reached laboratories remotely using technologies such as Zoom, WhatsApp, and Facebook Messenger.

Modeled on the ECHO project^11^ but adapted independently by the project team for laboratory mentoring, Zoom video conferencing technology was used to connect with the cohort of laboratories weekly (though often 2 or 3 times per week, on-demand) in a community of practice environment. Weekly training sessions followed a structured training schedule designed over a period of 16 months. This schedule was organized into weekly topics and followed a format of teacher presentation, laboratory presentation, question and answer sessions, and action items for the following week. Time for peer networking was also provided, and conversations on Zoom often carried over into other platforms such as Messenger and WhatsApp in the days following each session. Remote training and mentoring sessions were designed to reach more geographically dispersed laboratory professionals without the limitations of resource-intensive travel, thus improving the cost-effectiveness of activities. Through the use of Zoom Pro accounts, project staff were able to schedule meetings for up to 100 participants for up to 2 hours, providing visual presentations and video demonstrations, with the added benefit that each session was recorded and available for later review by participants. These trainings were designed primarily for QAOs and laboratory managers; however, all laboratory staff were welcome and many additional staff also attended the weekly sessions, with each session recording up to 28 participants from the combined group of laboratories. Importantly, the program enjoyed strong engagement from MOH-BMLS, which was involved in all project planning, implementation, and monitoring including all formal training sessions, workshops, and audits. This involvement was essential, ensuring the continuity and sustainability necessary for the program to be replicated within other laboratories once current funding had ended. Following this 16-month period of training and mentoring, a second CamLQMS audit was conducted and these data, along with an assessment of activity outputs, are the foundation of this study and program evaluation.

Remote training and mentoring sessions were designed to reach more geographically dispersed laboratory professionals without the limitations of resource-intensive travel.

## METHODS

### Research Methodology

This study evaluated the outputs and outcomes of Phase 2 activities in the 12 participating laboratories during the evaluation period of January 2018 to April 2019 between program audits. Our evaluation used an uncontrolled longitudinal study to assess changes in LQMS compliance to international standards between baseline and endpoint measurements. A cross-sectional analysis was then used to compare postimplementation LQMS performance and conformity of intervention laboratories to a select group of nonintervention laboratories. Data management and basic descriptive statistics for all evaluation methods were performed using Microsoft Excel for Office 365. All complex calculations of statistics and hypothesis testing were performed using STATA 14 statistical software.

### Quantifying Mentoring and Training Activity Outputs

For the description and enumeration of activity outputs, this study used monitoring and evaluation records collected by the project team. Outputs of interest, as listed in the logic model in Table 1, included (1) the number of trainings attended by personnel of the intended audience per laboratory, (2) the number of days of on-site mentorship provided to each laboratory, and (3) the amount of video conference training and mentoring time attributed to individual laboratory personnel during the evaluation period. Data sources included (1) attendance records for the 11 completed trainings, (2) project team member reports, (3) Zoom meeting records extracted from project team member’s Zoom accounts (reviewed to match laboratory and position details of meeting participants to their Zoom user names), and (4) supplementary records of remote mentoring sessions conducted via Messenger and WhatsApp from mentors. Datasets from each of these data sources were organized into separate spreadsheets for review and descriptive analysis. Attendance records for all 11 training events were organized by meeting date, and participant data were analyzed for each training event, which generally included laboratory managers and QAOs but at times included directors or administrators, biosafety officers, equipment officers, or stock officers. Counts were calculated by laboratory and event; the sum and average were calculated for the group. Records of on-site mentoring were similarly analyzed using mentoring reports as primary data sources. Both the number of participants per training and the number of in-person visits were planned and expected to be approximately equal between laboratories. Laboratories were allotted an equal number of days of on-site mentorship, although some training content varied based on laboratory need. Meanwhile, scheduled video conference training and mentoring were more client driven and scheduled meetings were provided on demand.

**TABLE 1.** Calculations of the 3 Primary Activity Outputs and the Cambodia Laboratory Quality Management System Audit Score Achieved Within the Evaluation Period

	No. Completed Trainings of Intended Participants (Total No. Participants)	Mentor Time on Site per Laboratory, Days	Video Conference Participation Time, Minutes	Audit Score Difference, %
Lab A	19 (25)	9	3,766	9
Lab B	21 (26)	10	5,855	13
Lab C	24 (25)	10	2,742	15
Lab D	23 (25)	10	6,320	32
Lab E	25 (29)	13	9,302	37
Lab F	24 (37)	13	9,664	31
Lab G	23 (24)	9	6,800	26
Lab H	21 (27)	13	5,290	28
Lab I	28 (36)	13	7,210	17
Lab J	22 (24)	8	4,434	28
Lab K	22 (26)	10	8,675	15
Lab L	22 (26)	12	2,263	7
Group mean ± SD	23±2 (28±4)	11±2	6,027±2,454	21±10

Because program mentors used a Zoom Pro account for most remote mentoring and training, meeting and participant data were automatically recorded through the report feature of the Zoom software and available for extraction and analysis. These reports were then compiled into a dataset including a join time, leave time, and a duration of participation (based entirely on duration of attendance) for each user identification (ID) during each meeting as a representative sample of remote mentoring activities. Within this dataset, attendance logs were tracked using participant IDs and crossmatched with participant work site/laboratory and job title, using mentor reports as supplemental records to match and attribute 98% of participation time to participating laboratory personnel, to project staff or mentors, or to other participating stakeholders. Due to the use of multiple devices by some participants during meetings, a dynamic Gantt chart was employed to visually and systematically identify duplicate, overlapping usernames. The duplicates were then recategorized as “device only” regarding position and laboratory to exclude them from analysis. All user logins that indicated multiple participants associated with a user ID were duplicated to reflect attendance of those participants. Minutes of participation time were grouped by laboratory and summarized for total participation time of unique attendees from each laboratory within the sample over the evaluation period. Records were then reviewed for additional remote training or mentoring events held outside of tracked video conferences to determine how representative the sample was out of the total estimate of events. Total video conference participation time per laboratory was then plotted in a scatter diagram against the percent differences in pre- and postintervention audit scores, described under the methods for CamLQMS outcome evaluation. Plots were visually inspected for a linear or monotonic relationship between the 2 variables and then tested for the strength of that relationship by using Spearman’s rank order correlation coefficient. Spearman’s rank was selected as a nonparametric test due to the small sample size of the intervention group (n=12), which was expected to increase the test sensitivity to moderate outliers in a Pearson’s test for correlation. Because formal trainings and site visits were restricted from random variation, our study was unable to provide similar correlation assessments between these activity outputs and direct program outcomes.

### Quantifying External Audit Outcomes

We used the CamLQMS checklist for accreditation as the primary outcome measure to determine the performance of each laboratory’s quality management system before and after the training and mentoring program interventions. The CamLQMS checklist was divided into 12 sections of laboratory quality with a total of 117 questions regarding whether a particular standard was met, and each question was assigned a numerical value that contributed to the audit score within each section and in the whole (Table 2). Mentored laboratories completed baseline CamLQMS audits in January of 2018 and outcome assessments in April of 2019. Additionally, a control group of representative public laboratories that did not receive LQMS training or mentoring (nonmentored/nonintervention) was selected for a cross-sectional comparison. Control laboratories were selected by MOH-BMLS as the nearest in comparable capacity in terms of the complementary package of activities and services, although these facilities differed significantly from mentored laboratories in terms of baseline level of training and number of staff. Laboratory audits of mentored and nonmentored facilities were conducted by 3 teams of auditors who were trained by the project team to assess facility conformity and nonconformity to the CamLQMS checklist. Each 4-person auditing team was led by a lead auditor and included at least 1 MOH-BMLS representative. During the audit process, the lead auditor asked each of the 117 questions of the laboratory in series, and the team reviewed responses at the end of each audit to determine whether the requirements of each question were met partially or in full, indicated by “yes,” “no,” or “partial.” Questions that were not applicable to a laboratory due to individual requirements or organizational complexities of the facility served were answered with “NA.” After completion of all audits, the 3 team leads reviewed all audit data together to identify any recording error, bias or inconsistencies in scoring methodologies between teams, and moderated audit point allocations accordingly. Audit scores were calculated as a percentage of the total value of checklist items for each section and overall for each laboratory.

**TABLE 2.** Cambodia Laboratory Quality Management System Checklist for Accreditation Score Sheet

Audit Score Sheet					
Section					**Total Points**
Section 1: Documents and Records					28
Section 2: Management Reviews					14
Section 3: Organization and Personnel					22
Section 4: Client Management and Customer Service					10
Section 5: Equipment					35
Section 6: Evaluation and Audits					15
Section 7: Purchasing and Inventory					24
Section 8: Process Control					32
Section 9: Information Management					21
Section 10: Identification of Nonconformities, Corrective, and Preventive Actions					19
Section 11: Occurrence/Incident Management and Process Improvement					12
Section 12: Facilities and Biosafety					43
Total score					275
Level 1 (0–150 pts) <55%	Level 2 (151–177 pts) 55%–64%	Level 3 (178–205 pts) 65%–74%	Level 4 (206–232 pts) 75%–84%	Level 5 (233–260 pts) 85%–94%	Level 6 (261–275 pts) ≥95%

A Wilcoxon signed-rank test for nonparametric comparison for paired samples was performed to determine the strength of the difference between 2018 and 2019 audit scores of mentored laboratories for each section and summary overall. Nonparametric statistics were selected to maintain consistent assumptions of normality between the groups of small sample size. Mean audit scores and standard deviations were calculated in each of the 12 sections for visual comparisons between laboratory groups, and all sections with statistically significant differences in scores between years were documented with the level of significance. The percent change in overall audit scores in each section was calculated to present the magnitude of change visually, and these percent differences were used as the primary variables for a Spearman’s rank correlation assessment of the relationship strength between audit score improvement and laboratory personnel participation time in Zoom activities. An assessment of the statistical difference between audit scores of mentored LQMS laboratories and nonmentored, non-LQMS comparison laboratories was performed using the Wilcoxon rank-sum test for 2 independent samples. Comparisons were made for overall audit scores and scores for individual audit sections, and all sections with statistically significant differences between groups were again documented.

## RESULTS

Table 1 shows the outputs for each measured program activity and the corresponding increase in CamLQMS audit score as the direct program outcome and reveals an output of 274 (mean=23±2) target personnel trainings, 72,321 (mean=6,027±2,454) minutes of video conference training, and 130 (mean=11±2) visits to laboratories, resulting in an average positive percent difference of 21±10% between the 2018 and 2019 overall audit scores. Video conference participation time was calculated from a sample size of 153 Zoom meetings with traceable usage reports out of a total of 261 meetings identified from supplemental mentor reports and program activity calendar entries. In terms of staff inputs, formal training and video conference activities included 2 primary mentors, 2 mentor trainees, the country project coordinator, and 3 laboratory systems technical and senior technical specialists. Additionally, several MOH officials from BMLS and the National Institute of Public Health participated in formal trainings and in numerous video conference activities.

**FIGURE 1.**
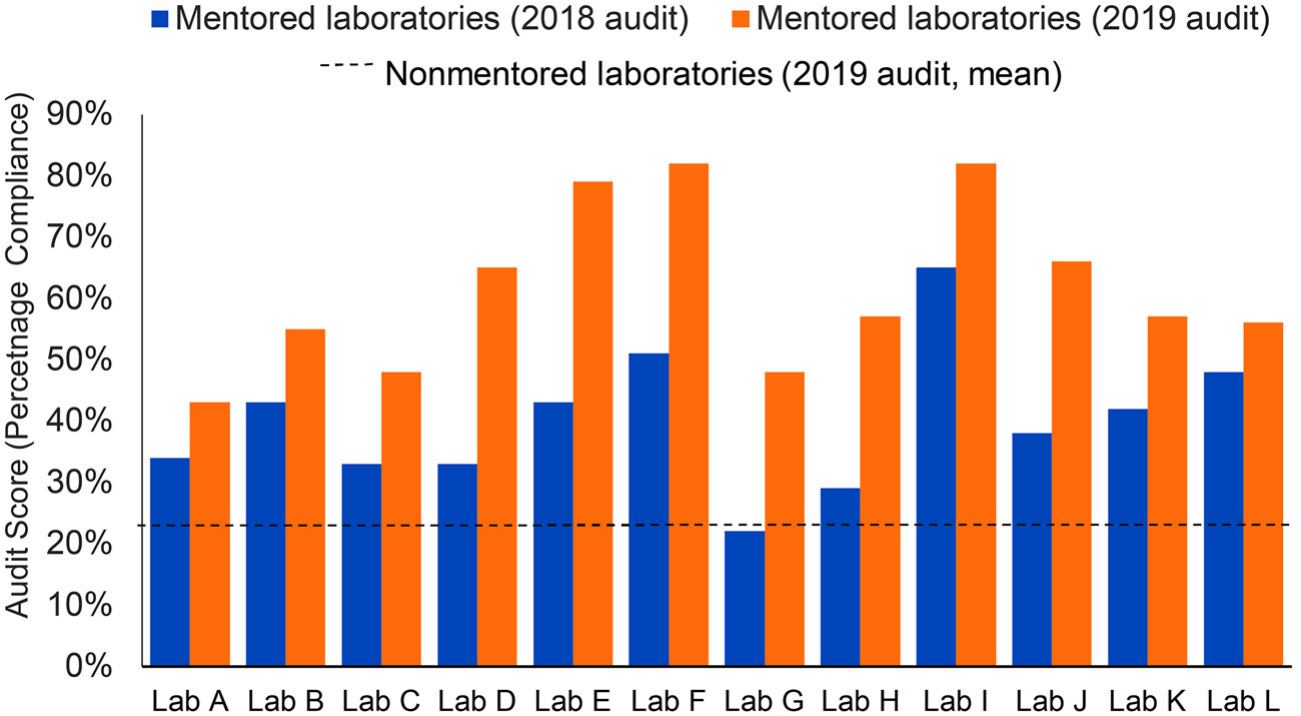
Overall 2018–2019 Cambodia Laboratory Quality Management System Checklist for Accreditation Audit Scores for Mentored Public Hospital Laboratories^a^ ^a^ The dashed line represents the average audit score for nonmentored laboratories (2018 audit data not available).

**FIGURE 2.**
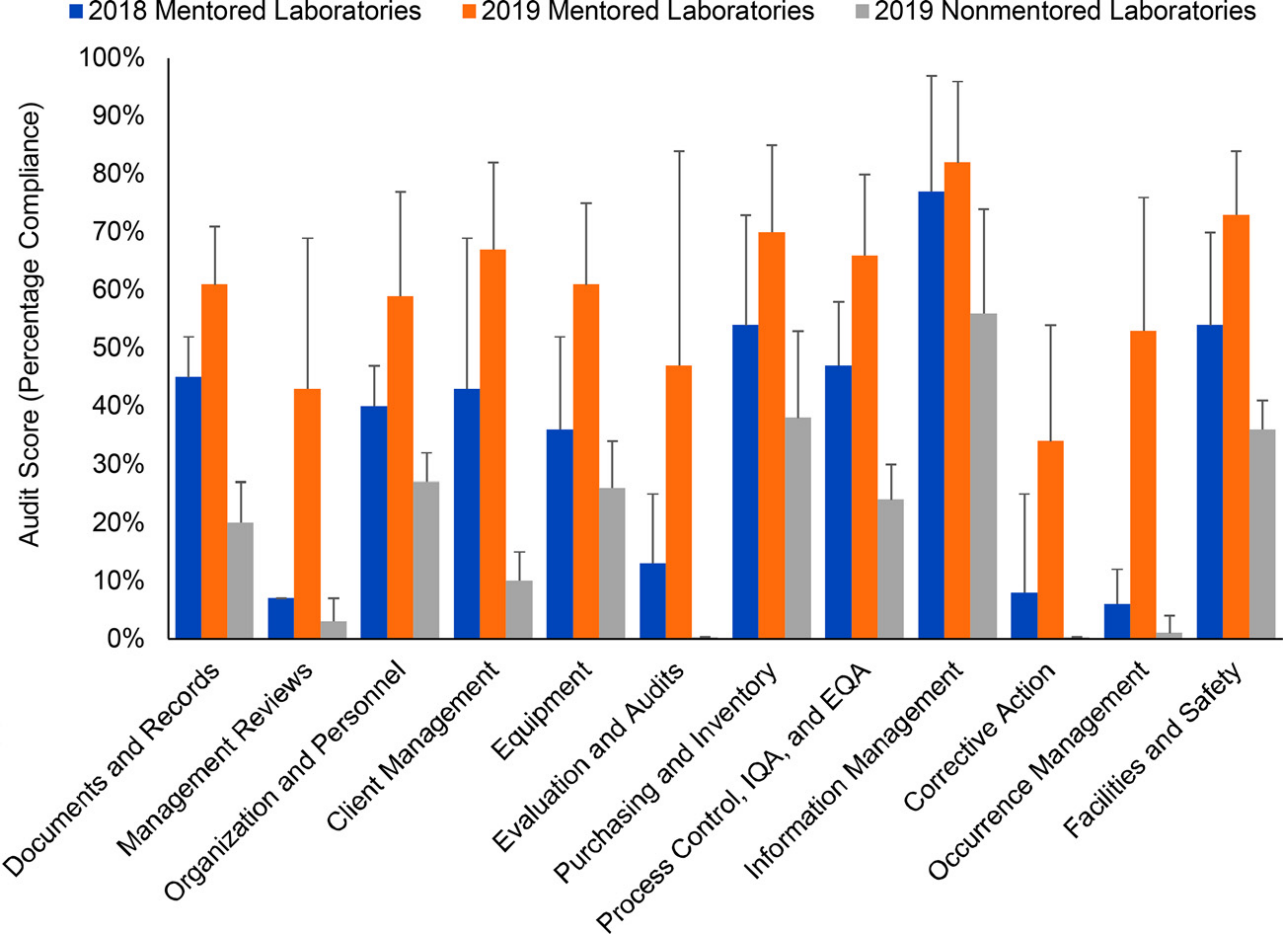
Mean Audit Scores of Mentored Public Hospital Laboratories Compared With Nonmentored Laboratories^a^ Abbreviations: EQA, external quality audit; IQA, internal quality audit. ^a^ Error bars represent the absolute standard deviations from the mean score of each section.

A Wilcoxon signed-rank test indicated that overall audit scores for mentored laboratories in 2019 were significantly higher (median score=57%) than overall audit scores for the same laboratories in 2018 (median score=40%, z=3.06, *P*=.002). In a comparison of scores for individual audit sections between years, the Wilcoxon signed-rank test indicated that mean 2019 scores for 11 out of 12 audit sections improved significantly (*P*<.01), with “information management” being the exception, which had been maintained but not significantly improved from an already high performance level at baseline (Figure 1). A cross-sectional comparison of the 2019 audit performance of mentored laboratories with the sample of nonmentored laboratories showed a large contrast in scores between groups (Figure 1) and by section (Figure 2).

A 2-sample Wilcoxon ranked-sum (Mann-Whitney) test indicated that overall audit scores for mentored laboratories in 2019 (median=57%) were significantly higher than overall audit scores for nonmentored laboratories (median=23%) in the same year (z=3.96, *P*=.0001). Mann-Whitney tests comparing individual audit sections similarly revealed significant differences in 11 of the 12 sections (*P*<.001) between intervention and nonintervention laboratories, with “information management” again being the exception, which was significantly different at the *P*<.01 level. In terms of percent difference in mean section scores between groups, “client management and customer service” as well as “occurrence management and process improvement” demonstrated the largest differences of 57% and 52% between groups. “Information management” again showed the smallest percent difference (27%) between groups. In an assessment of the relationship between mean audit score differences from 2018 to 2019 and the amount of participation time by individual laboratories in Zoom video conference training, a Spearman’s rank correlation showed a strong monotonic relationship between the 2 variables (r_s_=0.59, *P*=0.04) with significant certainty.

## DISCUSSION

The quality audit scores of laboratories participating in this program improved significantly following implementation of the training and mentoring activities, demonstrating that the QI program achieved its intended effect. Laboratory performance from mentored sites was significantly higher in all measured categories of quality management than in laboratories with no training or mentoring support, and this study clearly showed a positive correlation between laboratory QI and participant contact time with trainers and mentors via remote mentoring. The strong correlation between remote mentoring through video conference calling and improved audit scores indicates remote mentoring is an effective QI tool and also presents a cost-effective alternative to on-site mentoring, which requires frequent travel to remote, hard-to-reach laboratories. A 1-week site visit from Phnom Penh to Kratie, for example, costs approximately US$398 for travel, lodging, and per diem for local mentors, but a Zoom Pro account can cost as low as US$45 *annually*. Although further studies are needed to evaluate the cost-effectiveness of remote versus on-site telementoring and other inputs such as on-site training or mentorship, our results suggest a notable cost-benefit of telementoring for LQMS improvement compared with on-site training. Remote mentoring has the further benefit of providing on-demand professional support and networking. A qualitative study of the remote mentoring program in Cambodia identified a number of recurring themes of benefits identified by participants, including that additional remote training reinforced concepts and provided peer learning opportunities and on-demand guidance; however, laboratories strongly preferred a more structured training format in the local language if online training was used.^19^ The use of video conferencing technologies for medical education and consultation shows promise as a tool to create communities of practice between laboratorians and other health practitioners in the future, a practice that will prove all the more valuable during the COVID-19 pandemic, given that online platforms have become the primary means of accessing professional training and consultation for many medical professionals.^20^ ^–^ ^22^

The quality audit scores of laboratories improved significantly, demonstrating that the QI program achieved its intended effect.

Notably, although attendance in formal, in-person training and the number of on-site visits were semi-controlled for variation and therefore could not be tested for a relationship to LQMS improvement, the relationship is expected. In particular, the content of the program’s formal training curriculum is reflected in several individual audit sections that demonstrated major improvements. Topics such as “documents and records,” “management review,” “occurrence management,” and “process control” received particular focus in formal trainings, and thus coincided with superior program outcomes. Of note, this program chose to deprioritize the topics of “information management” and “facilities and safety” due to topical overlap with other ongoing national training programs. During site visits, mentors worked closely with laboratories on specific technical needs such as improved use of quality indicators (“occurrence management”), quality control testing (“process control”), “equipment” verification, and “corrective action.” In later stages of the implementation period, mentors coached personnel on internal auditing in preparation for the second round of CamLQMS audits. This close mentoring approach was predicted to have contributed to the program outcomes; however, further research is needed to isolate the impact of our program’s site visit and formal training models from that of remote mentoring.

Notable limitations and recommendations for future research are as follows. First, the CamLQMS checklist for accreditation is designed to assess gaps in conformity within individual laboratories to drive improvement in each specific section of LQMS. Because individual audit sections have different maximum possible scores, and because some questions are inapplicable to certain laboratories, overall audit comparisons between laboratories and sections should be interpreted with caution. Nonetheless, these comparisons serve as a useful estimate of program activity efficacy. Second, because our cross-sectional comparison of final CamLQMS results in program laboratories with nonprogram laboratories does not compare rates of change between groups, further prospective studies are needed to compare the rate of improvement directly through a pre-post design with a larger sample of facilities. The comparison group is also limited in its usefulness because of critical differences in staff size and training input at baseline. Control laboratories had 4–8 employees per facility compared with approximately 9–33 employees in the participant group and did not benefit from Phase 1 inputs, which resulted in better audit scores at baseline for mentored laboratories and may have provided a learning advantage over nonintervention laboratories. Finally, because monitoring of Zoom session reports was incorporated late into the program evaluation, 2% of participation time in video conferencing could not be associated with or disassociated from individual laboratories, leaving the potential of misclassification bias against certain laboratories prone to using unidentified devices. Built-in user report tools such as within Zoom serve as an easy-to-use mechanism for monitoring and evaluation of remote training and mentoring programs; however, some effort is needed to ensure data quality as it is collected.

Conventional in-training programs are resource intensive; however, as we have described here, programs that use remote mentoring and training tools such as Zoom can circumvent the need for frequent activities that are high-cost elements such as on-site workshops and coaching. In addition, they have the added benefit of reaching a larger audience than would otherwise be possible due to cost. These findings contribute to the limited body of qualitative research on remote mentoring as a practice, which describes success in QI outcomes due to improved accountability, collaborative problem solving, and increased awareness of the importance of laboratory quality.^13^ This evaluation strongly suggests that tiered laboratory systems in resource-limited countries such as Cambodia would benefit from national expansion of LQMS training and mentorship programs of a similar design, at scale, utilizing a structured curriculum and particularly remote training and mentoring methodologies.

## CONCLUSION

This program used a combination of training, mentoring, and advocacy to achieve rapid and significant outcomes in quality management system development. Participating laboratories performed significantly better in audits of performance and conformity than nonintervention laboratories, suggesting that an expansion of this methodology in Cambodia may benefit currently nonmentored laboratories significantly toward meeting national standards of quality. Although our findings indicate that significant progress has been made in meeting international standards of quality in laboratory practice, laboratories in the public sector and laboratories in Cambodia should continue to implement stepwise QI programs with an emphasis on improved connectivity of laboratories to professional training and mentorship for effective QI.

## Notes

**Peer Reviewed**

**First published online:** December 11, 2020

**Funding:** This work was funded by the U.S. Department of Defense, Defense Threat Reduction Agency and the views of the authors do not represent those of the funder.
