# Corrigendum: Donovan et al., Remote Mentorship Using Video Conferencing as an Effective Tool to Strengthen Laboratory Quality Management in Clinical Laboratories: Lessons From Cambodia

**DOI:** 10.9745/GHSP-D-21-00311

**Published:** 2021-06-30

See corrected article.

In the article “Remote Mentorship Using Video Conferencing as an Effective Tool to Strengthen Laboratory Quality Management in Clinical Laboratories: Lessons From Cambodia” by Grant Donovan et al., which appeared in the December 2020 issue (Volume 8, Issue 4), Patricia Sadate-Ngatchou is now listed as an author and Malin Chou is now added to the Acknowledgments for their respective contributions to the article.

The article has been corrected accordingly.

## Notes

Published: June 30, 2021.

